# Effectiveness of Intravitreal Dexamethasone Implant Treatment for Diabetic Macular Edema in Vitrectomized Eyes

**DOI:** 10.4274/tjo.galenos.2019.95226

**Published:** 2019-12-31

**Authors:** Hüseyin Baran Özdemir, Murat Hasanreisoğlu, Murat Yüksel, Mestan Ertop, Gökhan Gürelik, Şengül Özdek

**Affiliations:** 1 Gazi University Faculty of Medicine, Department of Ophthalmology, Ankara, Turkey

**Keywords:** Diabetic macular edema, DMÖ, dexamethasone implant, pars plana vitrectomy

## Abstract

**Objectives:**

To report the effectiveness and long-term outcomes of intravitreal dexamethasone implantation for diabetic macular edema (DME) in vitrectomized eyes

**Materials and Methods:**

Medical records of patients were retrospectively reviewed. Time of pars plana vitrectomy (PPV), PPV indications, interval between DEX injection and PPV, other intravitreal treatment prior to DEX application, best corrected visual acuity (BCVA), intraocular pressure (IOP), and central retinal thickness (CRT) measured by optical coherence tomography were recorded.

**Results:**

Seventeen eyes of 17 patients were included in the study. The mean follow-up after DEX injection was 21±2.4 months (12-43 months). The female/male ratio was 11/6. Mean age was 60.7 years (46-70 years). Sixteen eyes (94.1%) were pseudophakic at the time of DEX treatment. The most common indication for PPV was tractional retinal detachment (8 eyes, 47.1%). Ten eyes (58.8%) received a single injection and a total of 30 DEX implantations were performed. Mean BCVA was 0.77 logarithm of the minimum angle of resolution (logMAR) units before the first injection and improved to 0.64, 0.68 and 0.66 logMAR after 1, 3 and 6 months, respectively (p<0.01). CRT decreased significantly from 452 µm at baseline to 310, 368±34 and 375 µm after 1, 3 and 6 months, respectively (p<0.04). Mean IOP was 16±1.2 mmHg at baseline and 18.2, 18.8 and 18.5 mmHg after 1, 3, and 6 months (p>0.05). Two eyes (%8) received topical anti-glaucoma medication (IOP≥25 mmHg). Similar results were observed in eyes receiving repeated DEX injections.

**Conclusion:**

Intravitreal DEX injection treatment seems to be effective for improving BCVA and decreasing CRT in vitrectomized eyes with DME. This effect seemed to last for 6 months in most eyes, but maximized at 3 months. Patients with repeated injections often require injection before 6 months.

## Introduction

Diabetic retinopathy is among the leading causes of blindness in developed societies.^1^ Patients with diabetic retinopathy often suffer from vision loss due to diabetic macular edema (DME).^2^ Although multiple factors play a role in the pathogenesis of DME, one of the main mechanisms involves the inflammatory pathway, which comprises many mediators such as vascular endothelial growth factor (VEGF), tumor necrosis factor-alpha, monocyte chemoattractant protein-1, and interleukin-1 beta.^3,4^ For this reason, intravitreal corticosteroids are often employed in the treatment of DME.^5,6,7,8^ Intravitreal dexamethasone and triamcinolone acetate are the corticosteroids most commonly used to treat DME.^9,10^

Despite therapeutic advances, diabetic patients may still require pars plana vitrectomy (PPV). The most common indications for PPV are vitreous hemorrhage due to proliferative diabetic retinopathy, tractional retinal detachment, and refractory DME.^11^ Intravitreal anti-VEGF and corticosteroid injections are often needed after PPV as well. Some reports have indicated that intravitreal drugs administered to vitrectomized patients have reduced half-life and efficacy.^12^

Slow-release intravitreal implants were introduced to the market with the aim of providing long-lasting intraocular drug activity.^13,14^ An intravitreal dexamethasone implant (DEX; Ozurdex, Allergan, Irvine, CA, USA) was developed for injection into the vitreous cavity and is indicated for DME.^15^ DEX, a biodegradable polymer composed of a combination of 0.7 mg dexamethasone and poly(lactic acid-co-glycolic acid), slowly degrades in the vitreous cavity to release DEX over a period of 6 months.^16^ This sustained-release feature of DEX is proposed to reduce the number of injections needed in vitrectomized eyes compared to other intravitreal treatments.

The aim of this study was to report the effectiveness and long-term outcomes of DEX used for the treatment of DME in vitrectomized eyes.

## Materials and Methods

This retrospective study included patients over 18 years of age who had previously undergone PPV surgery in the Ophthalmology Department of Gazi University and who were subsequently given DEX injections due to DME between July 2015 and December 2017. The study was approved by a local ethics committee (Numune Training and Research Hospital Ethics Committee, decision E-19-2466) and conducted in adherence with the principles of the Declaration of Helsinki. Patients with less than 1 year of follow-up after DEX injection were not included in the study.

The patients were evaluated in terms of age; sex; affected eye; date and reason for PPV surgery; number of DEX injections; time interval between injections, if applicable; complications; total follow-up time; and best corrected visual acuity (BCVA), intraocular pressure (IOP), anterior segment examination findings (especially lens status), fundus examination findings, and central foveal thickness (CFT) obtained by optical coherence tomography (OCT) before and at 1, 3, and 6 months after injection. BCVA values were obtained with Snellen chart and converted from decimal to logarithm of the minimum angle of resolution (logMAR) before statistical analysis. CFT measurements in OCT (Spectralis OCT, Heidelberg Engineering, Heidelberg, Germany) were made using the values acquired automatically by the device.

### Statistical Analyses

SPSS software (version 22.0, SPSS, Inc. Chicago, IL, USA) was used for statistical analysis. Kolmogorov-Smirnov test was used to determine whether the data showed normal distribution. A Wilcoxon signed-rank test was used to evaluate changes in BCVA, IOP, and CFT values between pre- and post-treatment time points. Changes with p values <0.05 were considered significant.

## Results

Seventeen eyes of 17 patients (11 women, 6 men) were included in the study. The demographic characteristics of the patients are shown in Table 1. Sixteen eyes (94.1%) were pseudophakic. Thirteen eyes (76.5%) had been treated with anti-VEGF before treatment with DEX. In all patients, the time between the last intravitreal anti-VEGF administration and DEX was at least 3 months, with a mean of 5.2±4.6 months (3-16 months). Ten eyes (58.8%) received a single DEX injection and 7 eyes (41.2%) received multiple injections. A total of 30 DEX injections were administered.

Mean BCVA was 0.77±0.35 logMAR before the first DEX injection and increased significantly to 0.64±0.33 (p=0.007), 0.68±0.08 (p=0.009), and 0.66±0.36 (p=0.016) at 1, 3, and 6 months after DEX injection, respectively. When patients who were treated with a single dose of DEX and those who required repeat DEX injection within 6 months or after more than 6 months were examined separately, it was observed that the change in final BCVA compared to initial BCVA was similar in all three subgroups (p=0.719). BCVA increased more than 2 rows in 7 eyes (41.1%).

Mean CRT was 452±97 µm before DEX injection and decreased significantly to 310±103 µm (p=0.001), 368±140 µm (p=0.004), and 375±125 µm (p=0.041) at 1, 3, and 6 months after treatment, respectively. Change in CRT between initial and final values was also statistically similar in subgroup analysis of patients treated with a single dose of DEX and those who required repeat DEX injection within 6 months or after more than 6 months (p=0.180). The reduction in CRT was maintained for 6 months in patients who required a single dose of DEX.

Mean IOP increased with the first DEX injection from an initial value of 16±3.6 mmHg to 18.2±3.88 (p=0.027), 18.8±1.8 (p=0.221), and 18.5±1.2 mmHg (p=0.285) at 1, 3, and 6 months after DEX treatment, respectively. Two eyes required topical antiglaucoma therapy (IOP>25 mmHg).

The only patient who was phakic at the beginning of follow-up developed nuclear cataract and underwent cataract surgery 10 months after a single DEX injection. No additional complications were observed. The median time between first and second DEX injections was 5 months (4-27 months). Of the 7 eyes that received another injection, 5 (71.4%) required the second dose of DEX within 6 months.

## Discussion

There is still debate regarding the agents to be used for the treatment of DME in diabetic eyes that have undergone PPV surgery. In the present study, a single DEX injection to vitrectomized eyes reduced CRT and improved vision compared to pre-treatment values for 6 months in more than half of the patients (10/17 eyes, 58.8%). Thirty percent of the eyes required a repeat injection before 6 months, and the treatment response in the eyes that received a second DEX injection (7/17 eyes, 41.2%) was similar to the results of the first DEX injection. These findings are consistent with previously published results.

The CHAMPLAIN trial by Boyer et al.^17^ was the first study to examine the outcomes of DEX injection in vitrectomized eyes. The results of this prospective study including 55 PPV patients followed for 26 weeks indicated that DEX injection was effective for the treatment of DME and had an acceptable safety profile. It was reported that DEX took effect within 1 week and reached maximum effect at 8 weeks. Shah et al.^18^ demonstrated that the activity of DEX in vitrectomized eyes increases over the first month and lasts for at least 3 months. We also observed maximum effect in the first month in the present study, but DEX activity lasted longer than 3 months in the majority of our patients and a single injection was sufficient for 58.8% of them.

The half-life of intravitreal drugs is associated with the presence of the vitreous. Most studies investigating the pharmacokinetics of intravitreal drugs in vitrectomized eyes were based on the results of animal experiments.^12^ Studies conducted in the eyes of macaque monkeys showed that anti-VEGF had a shorter half-life in vitrectomized eyes.^19,20^ In studies of rabbit eyes, it was reported that the pharmacokinetics of ranibizumab and bevacizumab do not differ between vitrectomized and nonvitrectomized eyes.^21,22^ Chin et al.^23^ reported that triamcinolone acetate clearance was accelerated in vitrectomized eyes. Similarly, the half-life of triamcinolone acetate has been shown to be shorter in vitrectomized eyes that undergo sub-Tenon’s injection.^24^ In a DEX study by Chang-Lin et al.^16^ comparing vitrectomized and nonvitrectomized rabbit eyes, the pharmacokinetic profile of DEX was similar in both groups.

Although animal studies give some insight into drug pharmacokinetics, they cannot provide exact information due to differences in vitreous volume compared to the human eye, and because animal studies generally involve lensectomy as well as vitrectomy and there is no pseudophakia model.^25^ Yanyalı et al.^26^ observed no significant clinical effect in vitrectomized eyes treated with bevacizumab due to DME. Studies on ranibizumab have shown that vitrectomized eyes require more injections compared to normal eyes for the treatment of DME, but there was no significant difference in terms of efficacy.^27,28^ The Diabetic Retinopathy Clinical Research Network (DRCR.net) group reported that favorable outcomes were obtained with ranibizumab in the vitrectomized eyes of patients who were followed for a mean of 3 years.^29^ In that study, it was reported that there was no significant difference between the two groups in terms of number of injections, but the clinical effect emerged more slowly and more injections were needed in the first year of treatment in vitrectomized eyes. The sustained-release DEX was reported to have similar pharmacokinetics in vitrectomized and nonvitrectomized rabbit eyes. As with anti-VEGF studies, most of the results from human eyes have been obtained from retrospective data.

Comparisons of the effectiveness of DEX in the treatment of DME in vitrectomized versus nonvitrectomized eyes in the literature have also been based on retrospective data. In their retrospective review of vitrectomized and nonvitrectomized groups including 24 eyes each, Medeiros et al.^30^ demonstrated that DEX had similar effectiveness in both groups in terms of visual improvement and decrease in CRT. Çevik et al.^31^ also reported that DEX was similarly effective in the treatment of DME in eyes with and without vitrectomy. Bastakis et al.^32^ reported that previous vitrectomy did not adversely affect the effectiveness of DEX in patients with refractory DME, and that the maximum effect was observed within the first 3 months in both vitrectomized and nonvitrectomized eyes.

### Study Limitations

The limitations of our study include its retrospective nature, the lack of a control group, and the small number of patients. Prospective, randomized studies should be expected to provide more accurate results when comparing the efficacy of DEX between eyes with and without previous vitrectomy. However, the results of comparative retrospective studies in the literature show that DEX can be used safely in patients with DME who have undergone vitrectomy. Strengths of our study are the long-term follow-up, the good collection of retrospective data, and the inclusion of real-life patient data.

## Conclusion

In conclusion, DEX is a safe and effective treatment for DME patients with history of PPV. DEX provides long-term vision increase and CRT decrease with a single injection in the majority of patients and shows a safe IOP profile, which suggests that it should be considered as first-line treatment in vitrectomized patients. It should be kept in mind that the effect may be shorter and that frequent injections may be necessary in patients with refractory DME.

## Figures and Tables

**Table 1:**
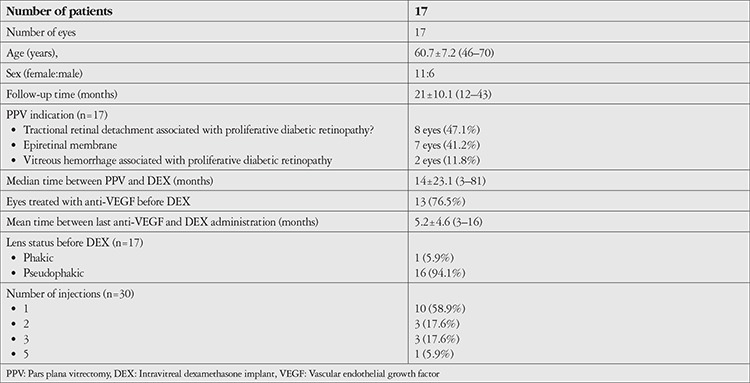
Demographic and clinical characteristics of the patients included in the study

## References

[1] Cheung N, Mitchell P, Wong TY (2010). Diabetic retinopathy. Lancet..

[2] Lee R, Wong TY, Sabanayagam C (2015). Epidemiology of diabetic retinopathy, diabetic macular edema and related vision loss. Eye Vis (Lond)..

[3] Das A, McGuire PG, Rangasamy S (2015). Diabetic Macular Edema: Pathophysiology and Novel Therapeutic Targets. Ophthalmology..

[4] Rangasamy S, McGuire PG, Das A (2012). Diabetic retinopathy and inflammation: novel therapeutic targets. Middle East Afr J Ophthalmol..

[5] Diabetic Retinopathy Clinical Research N, Wells JA, Glassman AR, Ayala AR, Jampol LM, Aiello LP, Antoszyk AN, Arnold-Bush B, Baker CW, Bressler NM, Browning DJ, Elman MJ, Ferris FL, Friedman SM, Melia M, Pieramici DJ, Sun JK, Beck RW (2015). Aflibercept, bevacizumab, or ranibizumab for diabetic macular edema. N Engl J Med..

[6] Wells JA, Glassman AR, Ayala AR, Jampol LM, Bressler NM, Bressler SB, Brucker AJ, Ferris FL, Hampton GR, Jhaveri C, Melia M, Beck RW;, Diabetic Retinopathy Clinical Research Network (2016). Aflibercept, Bevacizumab, or Ranibizumab for Diabetic Macular Edema: Two-Year Results from a Comparative Effectiveness Randomized Clinical Trial. Ophthalmology..

[7] Nurözler Tabakcı B, Ünlü N (2017). Corticosteroid Treatment in Diabetic Macular Edema. Turk J Ophthalmol..

[8] Akıncıoğlu D, Küçükevcilioğlu M, Durukan AH, Aykaş S, Ayyıldız Ö, Erdurman FC (2017). Outcomes of Intravitreal Dexamethasone Implant in the Treatment of Recalcitrant Diabetic Macular Edema. Turk J Ophthalmol..

[9] Yang Y, Bailey C, Loewenstein A, Massin P (2015). Intravitreal Corticosteroids in Diabetic Macular Edema: Pharmacokinetic Considerations. Retina..

[10] Lattanzio R, Cicinelli MV, Bandello F (2017). Intravitreal Steroids in Diabetic Macular Edema. Dev Ophthalmol..

[11] Gupta V, Arevalo JF (2013). Surgical management of diabetic retinopathy. Middle East Afr J Ophthalmol..

[12] Edington M, Connolly J, Chong NV (2017). Pharmacokinetics of intravitreal anti- VEGF drugs in vitrectomized versus non-vitrectomized eyes. Expert Opin Drug Metab Toxicol..

[13] Zucchiatti I, Lattanzio R, Querques G, Querques L, Del Turco C, Cascavilla ML, Bandello F (2012). Intravitreal dexamethasone implant in patients with persistent diabetic macular edema. Ophthalmologica..

[14] Campochiaro PA, Brown DM, Pearson A, Ciulla T, Boyer D, Holz FG, Tolentino M, Gupta A, Duarte L, Madreperla S, Gonder J, Kapik B, Billman K, Kane FE;, FAME Study Group (2011). Long-term benefit of sustaineddelivery fluocinolone acetonide vitreous inserts for diabetic macular edema. Ophthalmology..

[15] Boyer DS, Yoon YH, Belfort R Jr, Bandello F, Maturi RK, Augustin AJ, Li XY, Cui H, Hashad Y, Whitcup SM;, Ozurdex MEAD Study Group (2014). Threeyear, randomized, sham-controlled trial of dexamethasone intravitreal implant in patients with diabetic macular edema. Ophthalmology.

[16] Chang-Lin JE, Attar M, Acheampong AA, Robinson MR, Whitcup SM, Kuppermann BD, Welty D (2011). Pharmacokinetics and pharmacodynamics of a sustained-release dexamethasone intravitreal implant. Invest Ophthalmol Vis Sci..

[17] Boyer DS, Faber D, Gupta S, Patel SS, Tabandeh H, Li XY, Liu CC, Lou J, Whitcup SM;, Ozurdex CHAMPLAIN Study Group (2011). Dexamethasone intravitreal implant for treatment of diabetic macular edema in vitrectomized patients. Retina..

[18] Shah AR, Xi M, Abbey AM, Yonekawa Y, Faia LJ, Hassan TS, Ruby AJ, Wolfe JD (2016). Short-term Efficacy of Intravitreal Dexamethasone Implant in Vitrectomized Eyes with Recalcitrant Diabetic Macular Edema and Prior Anti-VEGF Therapy. J Ophthalmic Vis Res..

[19] Niwa Y, Kakinoki M, Sawada T, Wang X, Ohji M (2015). Ranibizumab and Aflibercept: Intraocular Pharmacokinetics and Their Effects on Aqueous VEGF Level in Vitrectomized and Nonvitrectomized Macaque Eyes. Invest Ophthalmol Vis Sci..

[20] Kakinoki M, Sawada O, Sawada T, Saishin Y, Kawamura H, Ohji M (2012). Effect of vitrectomy on aqueous VEGF concentration and pharmacokinetics of bevacizumab in macaque monkeys. Invest Ophthalmol Vis Sci..

[21] Ahn J, Kim H, Woo SJ, Park JH, Park S, Hwang DJ, Park KH (2013). Pharmacokinetics of intravitreally injected bevacizumab in vitrectomized eyes. J Ocul Pharmacol Ther..

[22] Ahn SJ, Ahn J, Park S, Kim H, Hwang DJ, Park JH, Park JY, Chung JY, Park KH, Woo SJ (2014). Intraocular pharmacokinetics of ranibizumab in vitrectomized versus nonvitrectomized eyes. Invest Ophthalmol Vis Sci..

[23] Chin HS, Park TS, Moon YS, Oh JH (2005). Difference in clearance of intravitreal triamcinolone acetonide between vitrectomized and nonvitrectomized eyes. Retina..

[24] Park HJ, Lee JE, Kim SI, Pak KY, Oum BS, Lee JS, Jung JH, Lee JE (2014). Intravitreal pharmacokinetics after posterior subtenon triamcinolone acetonide injection in vitrectomized rabbit eyes. Retina..

[25] Kerimoğlu H (2018). Intravitreal Anti-VEGF Drug Injections in Vitrectomized Eyes. Ret-Vit..

[26] Yanyali A, Aytug B, Horozoglu F, Nohutcu AF (2007). Bevacizumab (Avastin) for diabetic macular edema in previously vitrectomized eyes. Am J Ophthalmol..

[27] Koyanagi Y, Yoshida S, Kobayashi Y, Kubo Y, Yamaguchi M, Nakama T, Nakao S, Ikeda Y, Ohshima Y, Ishibashi T, Sonoda KH (2016). Comparison of the Effectiveness of Intravitreal Ranibizumab for Diabetic Macular Edema in Vitrectomized and Nonvitrectomized Eyes. Ophthalmologica..

[28] Chen YY, Chen PY, Chen FT, Chen YJ, Wang JK (2018). Comparison of efficacy of intravitreal ranibizumab between non-vitrectomized and vitrectomized eyes with diabetic macular edema. Int Ophthalmol..

[29] Bressler SB, Melia M, Glassman AR, Almukhtar T, Jampol LM, Shami M, Berger BB, Bressler NM;, Diabetic Retinopathy Clinical Research Network (2015). Ranibizumab Plus Prompt or Deferred Laser for Diabetic Macular Edema in Eyes with Vitrectomy before Anti-Vascular Endothelial Growth Factor Therapy. Retina..

[30] Medeiros MD, Alkabes M, Navarro R, Garcia-Arumi J, Mateo C, Corcostegui B (2014). Dexamethasone intravitreal implant in vitrectomized versus nonvitrectomized eyes for treatment of patients with persistent diabetic macular edema. J Ocul Pharmacol Ther..

[31] Çevik SG, Yılmaz S, Çevik MT, Akalp FD, Avcı R (2018). Comparison of the Effect of Intravitreal Dexamethasone Implant in Vitrectomized and Nonvitrectomized Eyes for the Treatment of Diabetic Macular Edema. J Ophthalmol..

[32] Bastakis GG, Dimopoulos D, Stavrakakis A, Pappas G (2019). Long-term efficacy and duration of action of dexamethasone implant, in vitrectomised and non-vitrectomised eyes with persistent diabetic macular oedema. Eye (Lond)..

